# Supplementary figures and images for: Structural and dynamic impacts of single-atom disruptions to guide RNA interactions within the recognition lobe of Geobacillus stearothermophilus Cas9
Source: eLife. 2025 May 19;13:RP99275. doi: 10.7554/eLife.99275 (PMC12088677; doi:10.7554/eLife.99275)

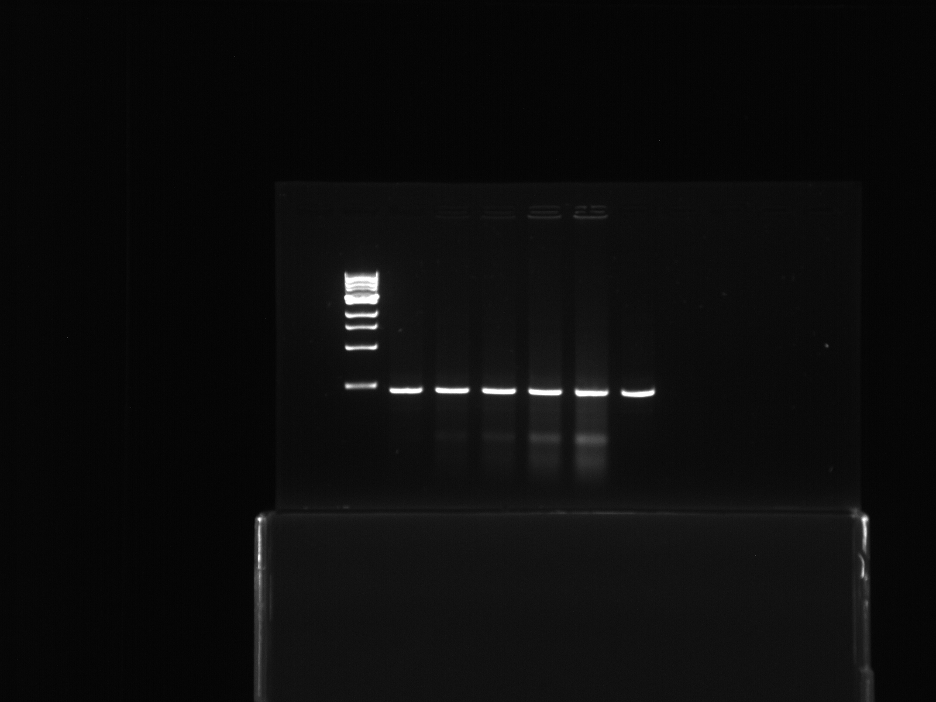

Supplement: Figure 7—figure supplement 1—source data 1. [file elife-99275-fig7-figsupp1-data1.zip › R332A GeoCas9/R332A GeoCas9 at 85 ┬░C.png]

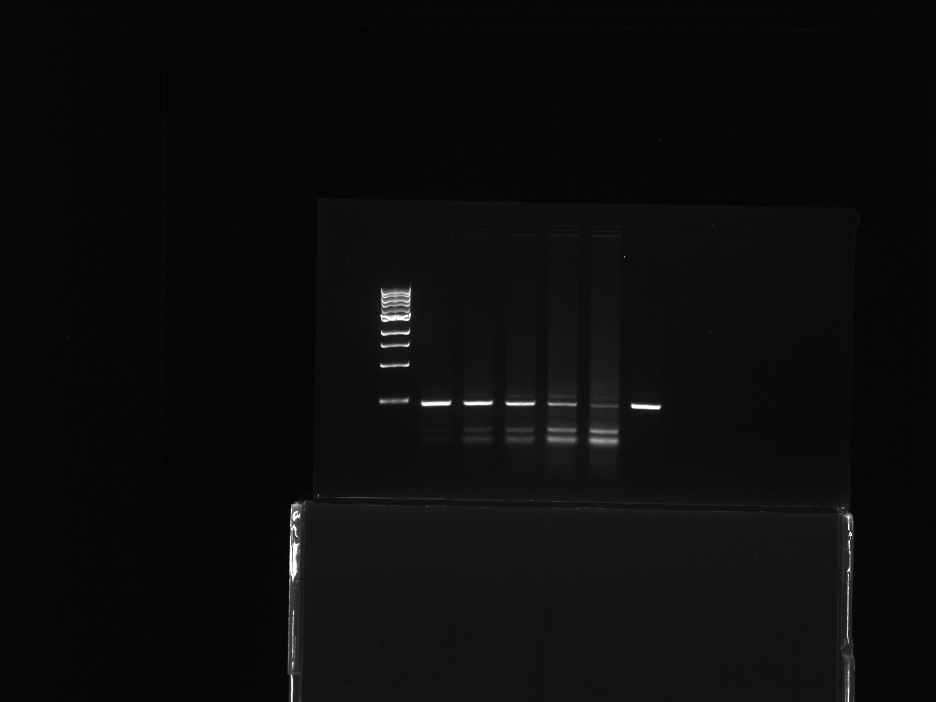

Supplement: Figure 7—figure supplement 1—source data 1. [file elife-99275-fig7-figsupp1-data1.zip › R332A GeoCas9/R332A GeoCas9 at 37 ┬░C.png]

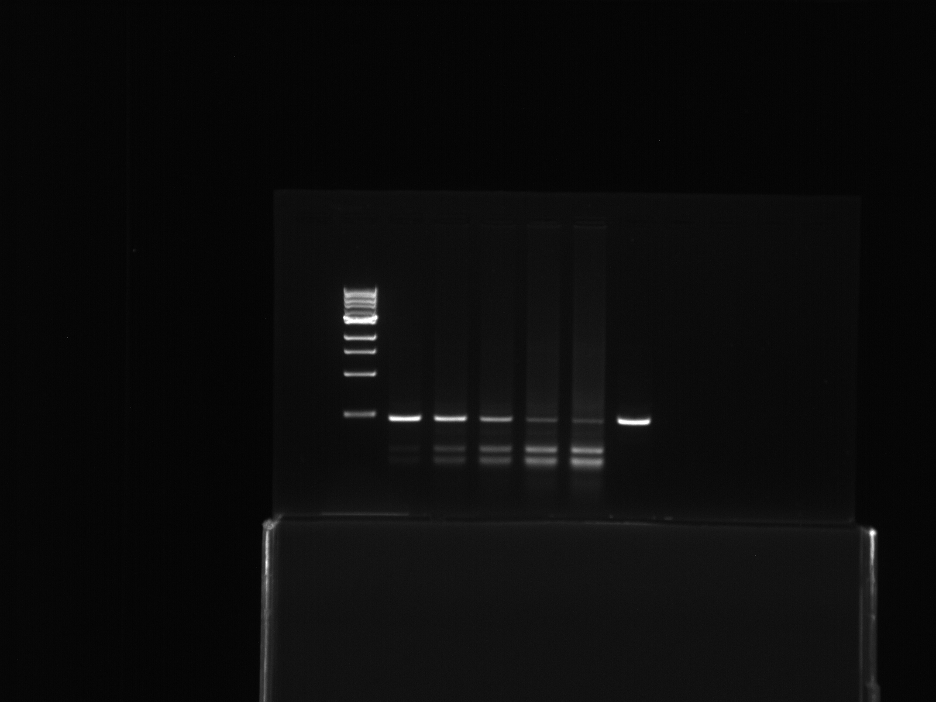

Supplement: Figure 7—figure supplement 1—source data 1. [file elife-99275-fig7-figsupp1-data1.zip › R332A GeoCas9/R332A GeoCas9 at 60 ┬░C.png]

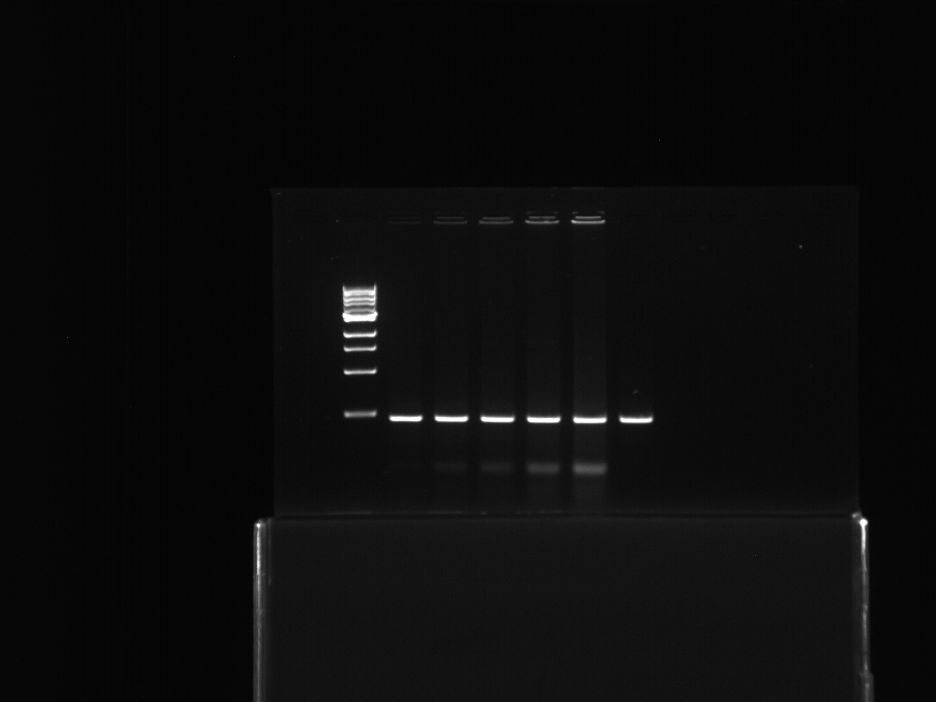

Supplement: Figure 7—figure supplement 1—source data 1. [file elife-99275-fig7-figsupp1-data1.zip › R332A GeoCas9/R332A GeoCas9 at 75 ┬░C.png]

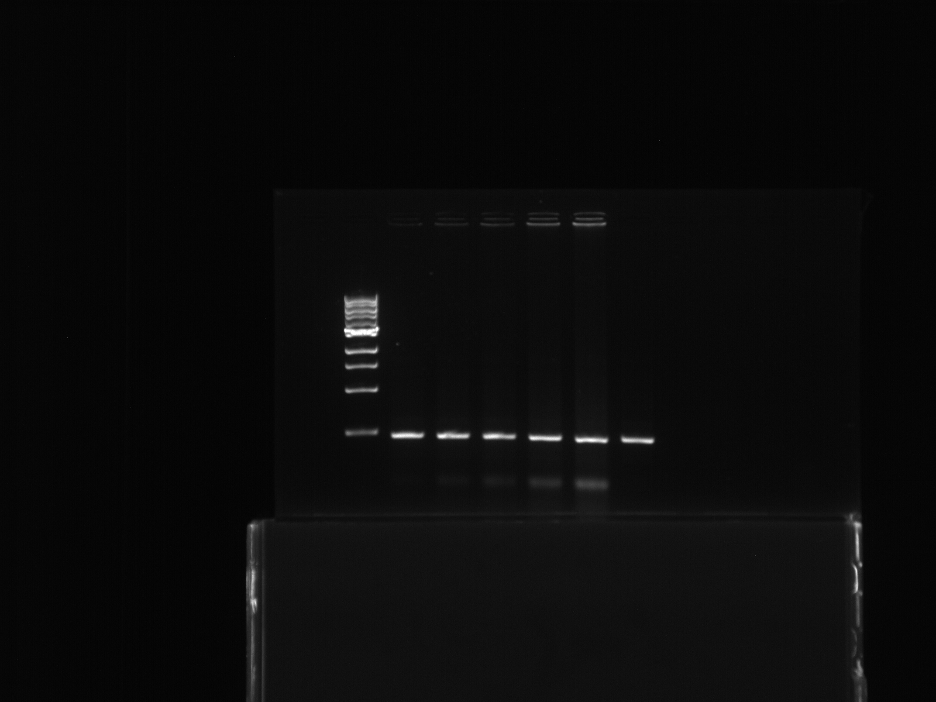

Supplement: Figure 7—figure supplement 1—source data 1. [file elife-99275-fig7-figsupp1-data1.zip › WT GeoCas9 /WT GeoCas9 at 75 ┬░C.png]

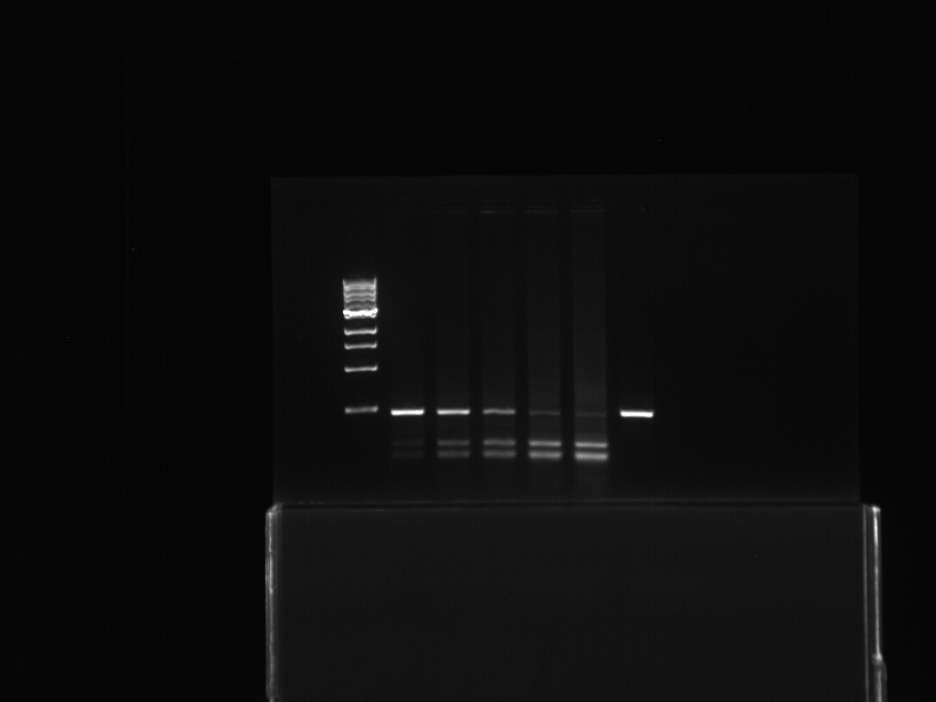

Supplement: Figure 7—figure supplement 1—source data 1. [file elife-99275-fig7-figsupp1-data1.zip › WT GeoCas9 /WT GeoCas9 at 60 ┬░C.png]

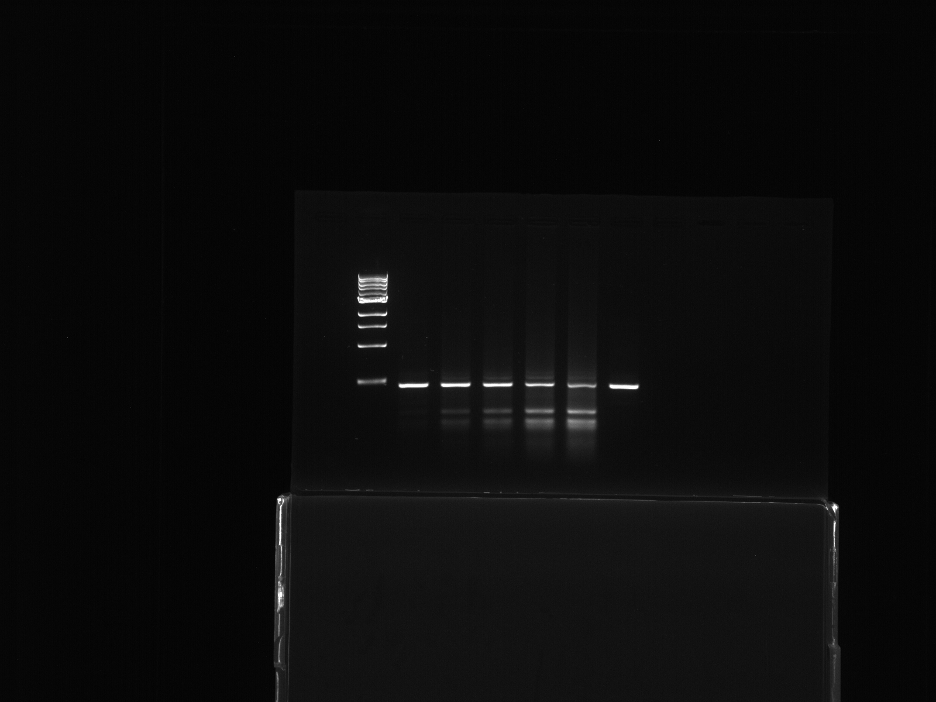

Supplement: Figure 7—figure supplement 1—source data 1. [file elife-99275-fig7-figsupp1-data1.zip › WT GeoCas9 /WT GeoCas9 at 37 ┬░C.png]

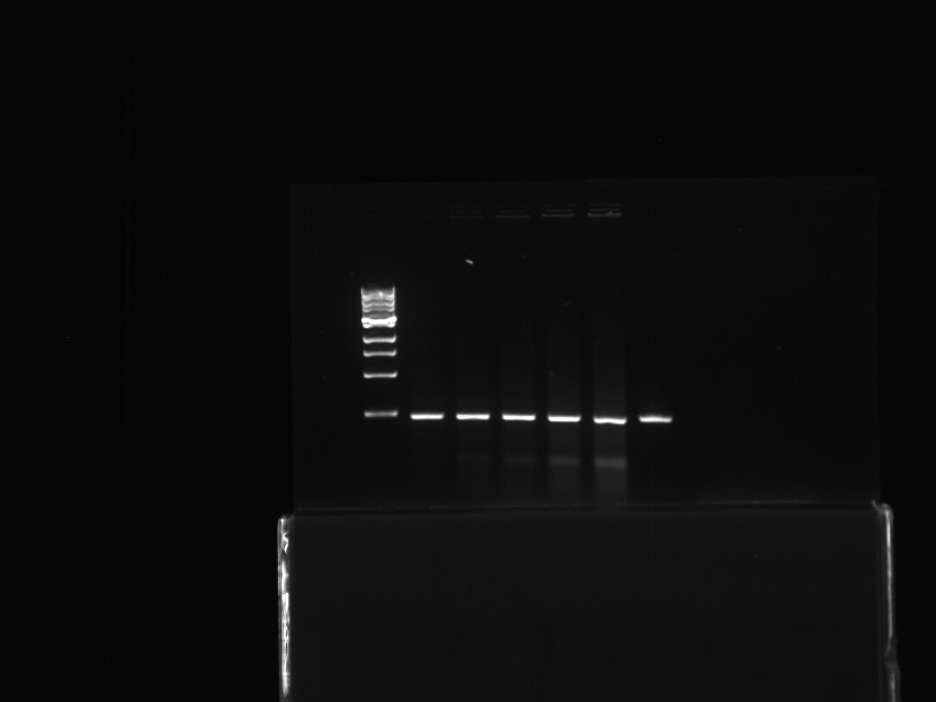

Supplement: Figure 7—figure supplement 1—source data 1. [file elife-99275-fig7-figsupp1-data1.zip › WT GeoCas9 /WT GeoCas9 at 85 ┬░C.png]

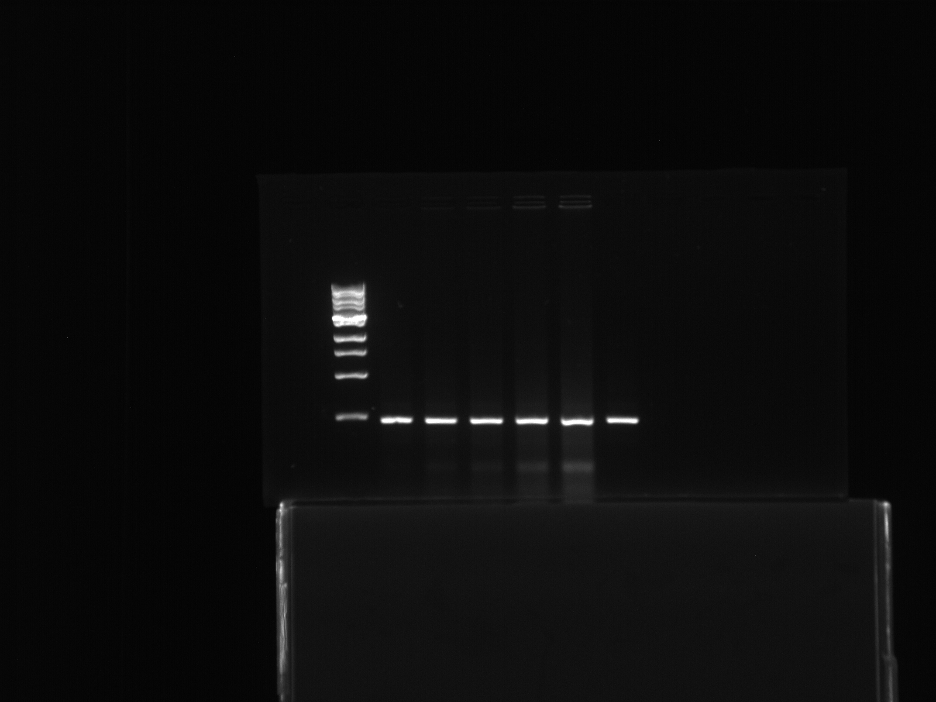

Supplement: Figure 7—figure supplement 1—source data 1. [file elife-99275-fig7-figsupp1-data1.zip › K267E GeoCas9/K267E GeoCas9 at 85 ┬░C.png]

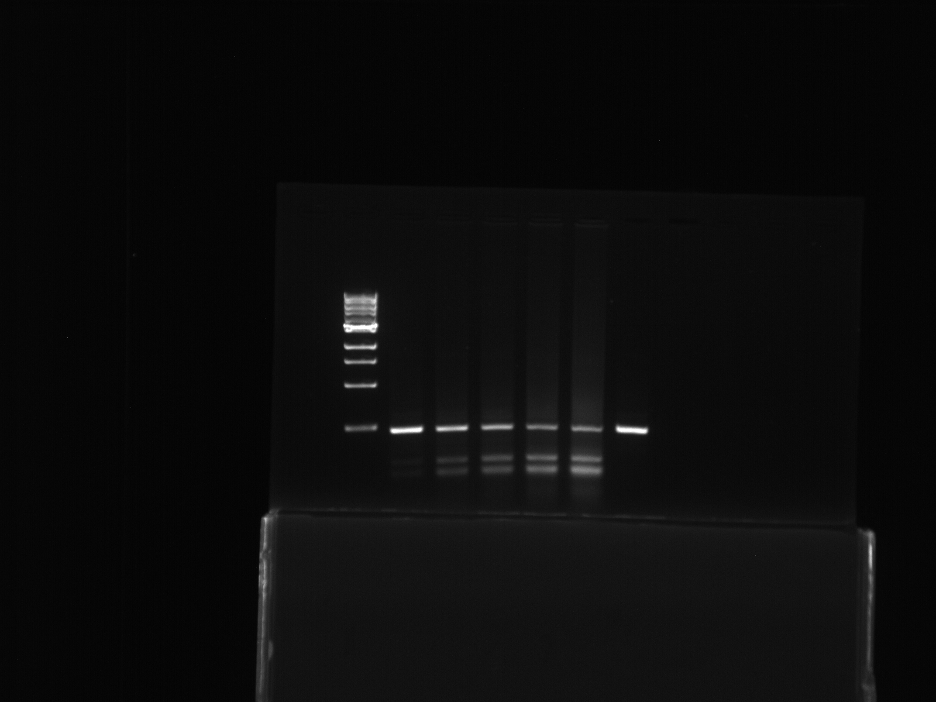

Supplement: Figure 7—figure supplement 1—source data 1. [file elife-99275-fig7-figsupp1-data1.zip › K267E GeoCas9/K267E GeoCas9 at 60 ┬░C.png]

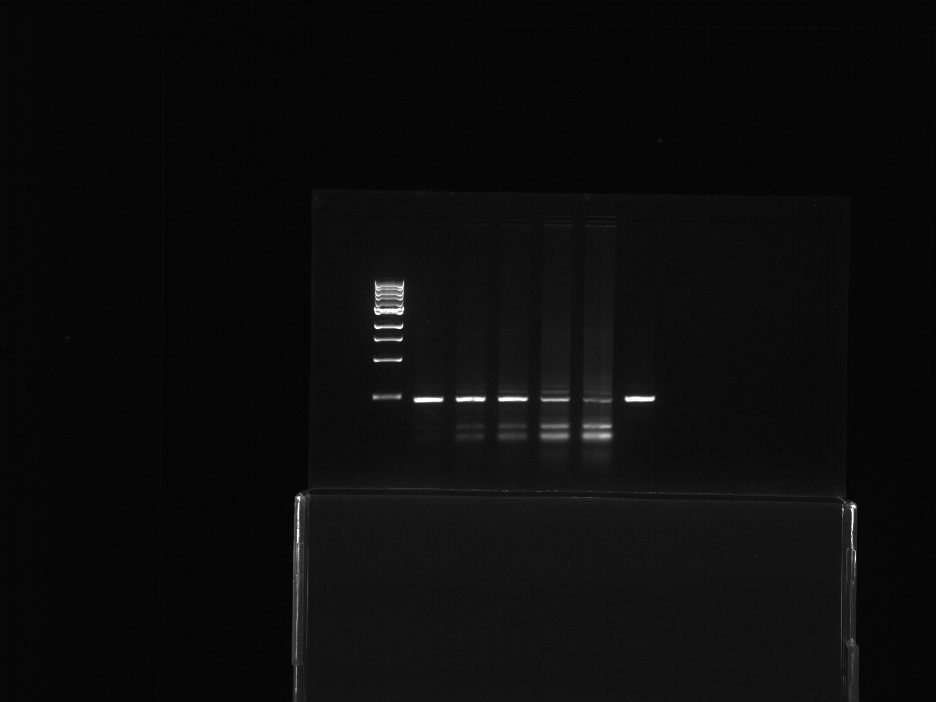

Supplement: Figure 7—figure supplement 1—source data 1. [file elife-99275-fig7-figsupp1-data1.zip › K267E GeoCas9/K267E GeoCas9 at 37 ┬░C.png]

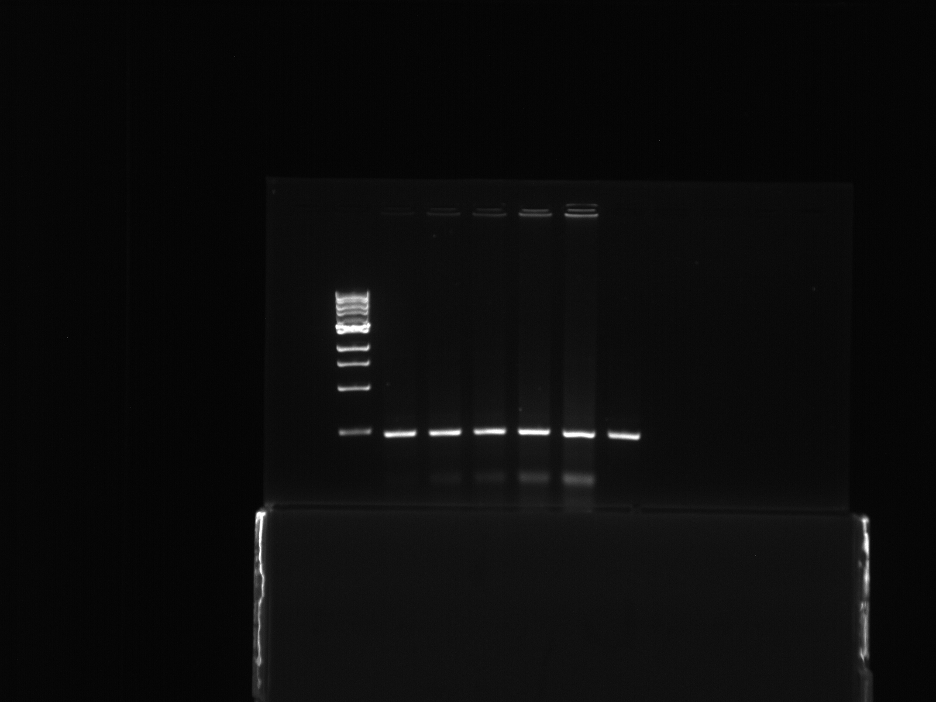

Supplement: Figure 7—figure supplement 1—source data 1. [file elife-99275-fig7-figsupp1-data1.zip › K267E GeoCas9/K267E GeoCas9 at 75 ┬░C.png]

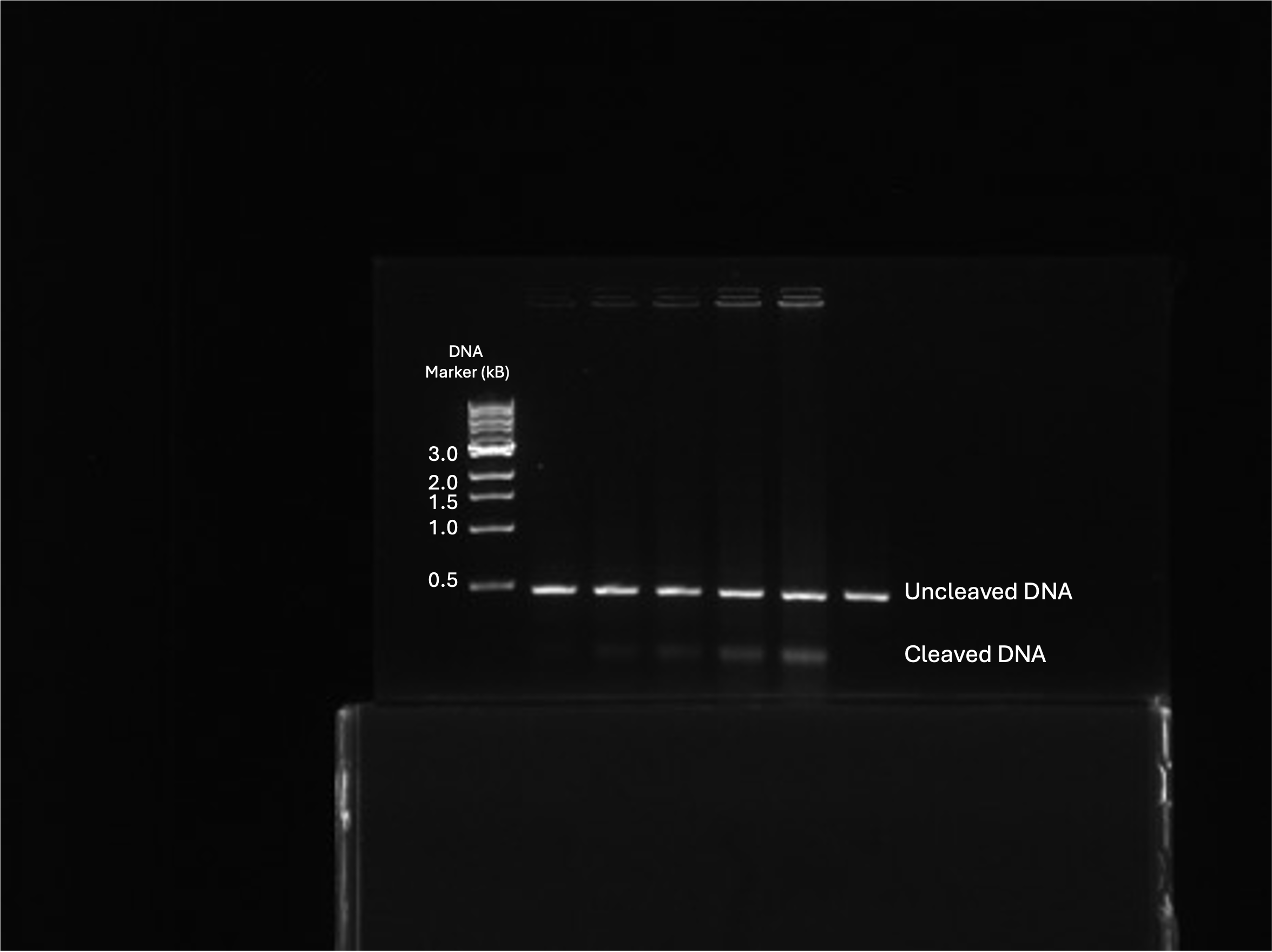

Supplement: Figure 7—figure supplement 1—source data 2. [file elife-99275-fig7-figsupp1-data2.zip › Figure 7 - figure supplement 1 - source data 2/WT GeoCas9 /WT GeoCas9 at 75 ┬░C.png]

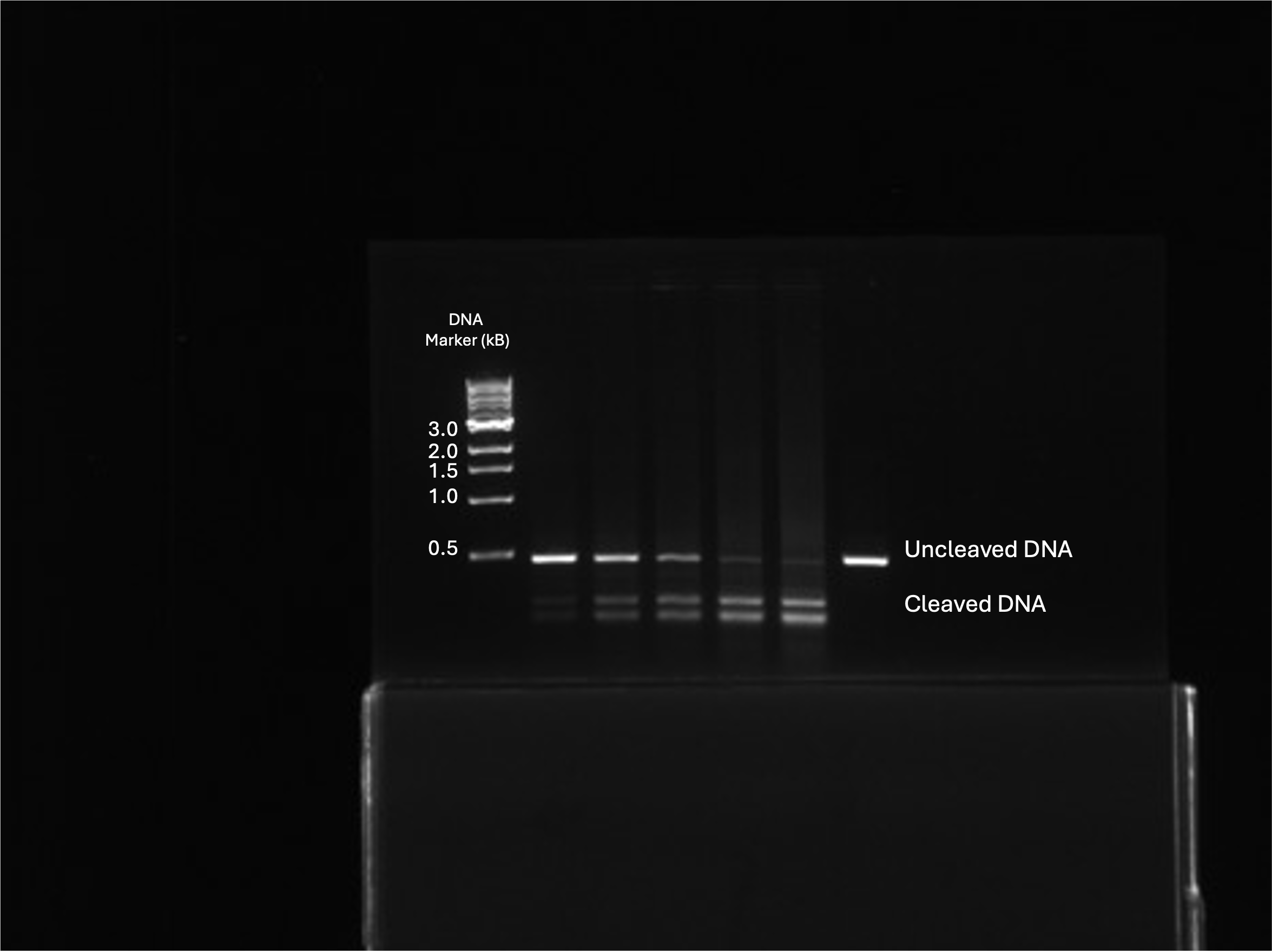

Supplement: Figure 7—figure supplement 1—source data 2. [file elife-99275-fig7-figsupp1-data2.zip › Figure 7 - figure supplement 1 - source data 2/WT GeoCas9 /WT GeoCas9 at 60 ┬░C.png]

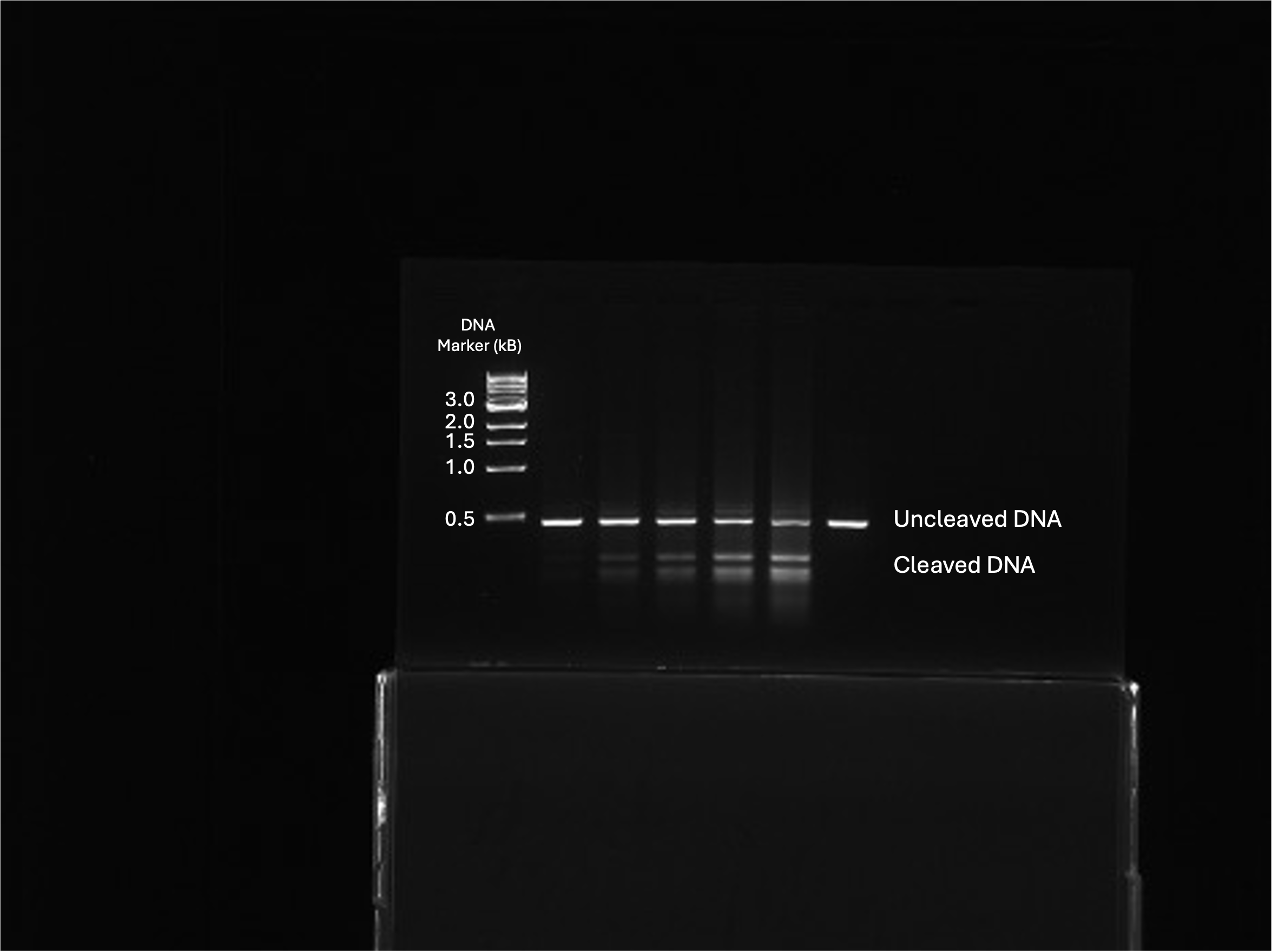

Supplement: Figure 7—figure supplement 1—source data 2. [file elife-99275-fig7-figsupp1-data2.zip › Figure 7 - figure supplement 1 - source data 2/WT GeoCas9 /WT GeoCas9 at 37 ┬░C.png]

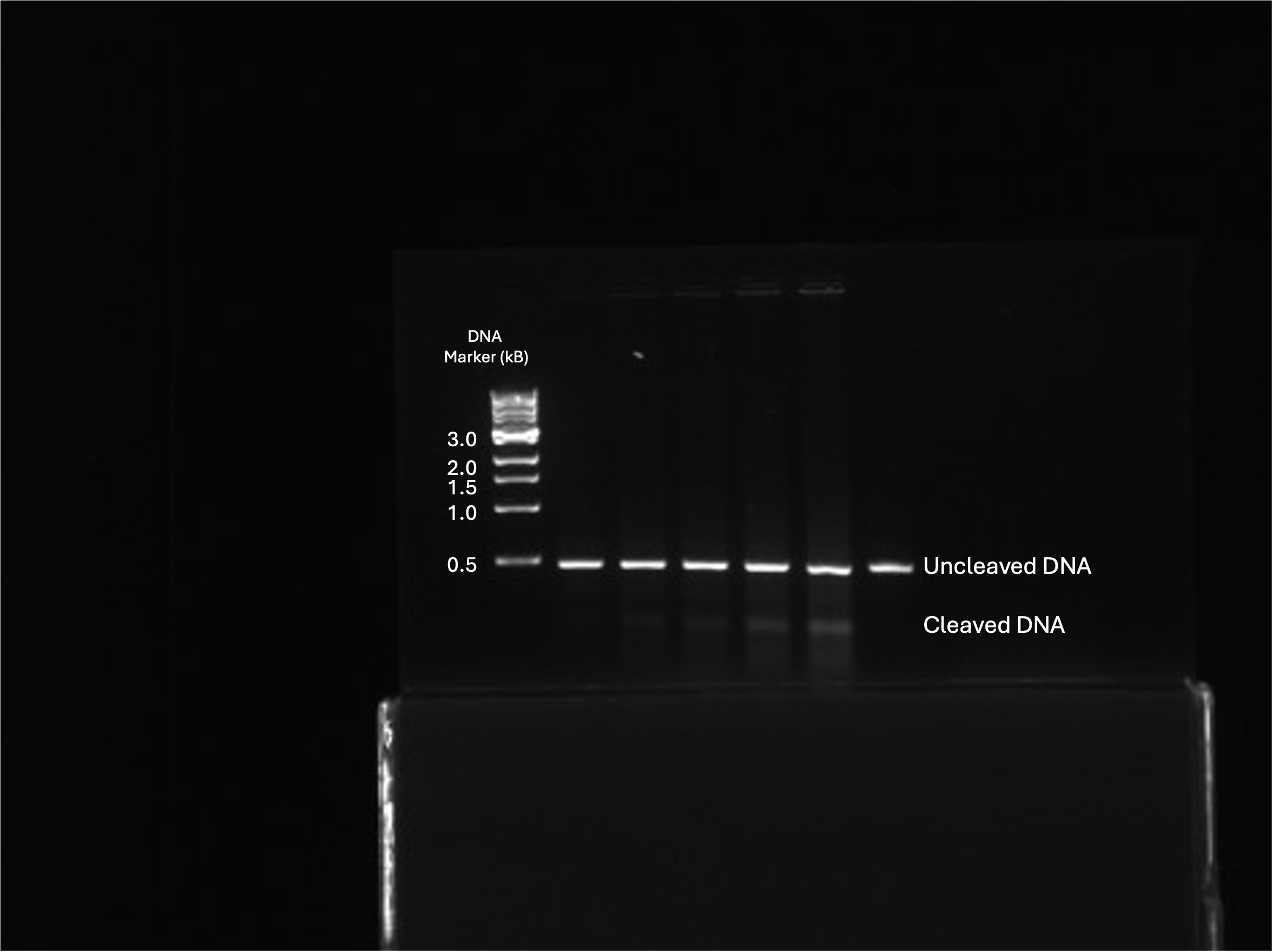

Supplement: Figure 7—figure supplement 1—source data 2. [file elife-99275-fig7-figsupp1-data2.zip › Figure 7 - figure supplement 1 - source data 2/WT GeoCas9 /WT GeoCas9 at 85 ┬░C.png]

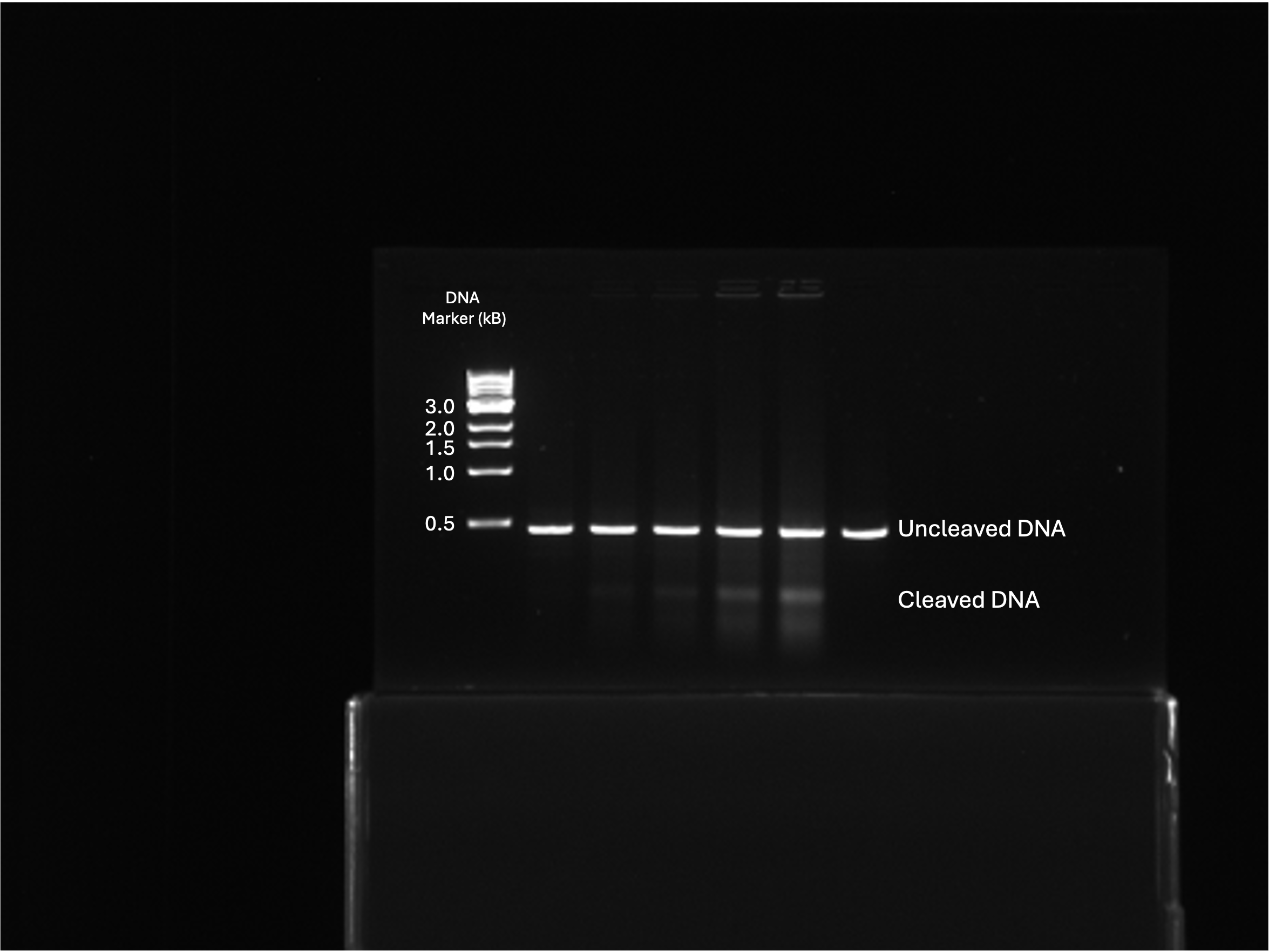

Supplement: Figure 7—figure supplement 1—source data 2. [file elife-99275-fig7-figsupp1-data2.zip › Figure 7 - figure supplement 1 - source data 2/R332A GeoCas9/R332A GeoCas9 at 85 ┬░C.png]

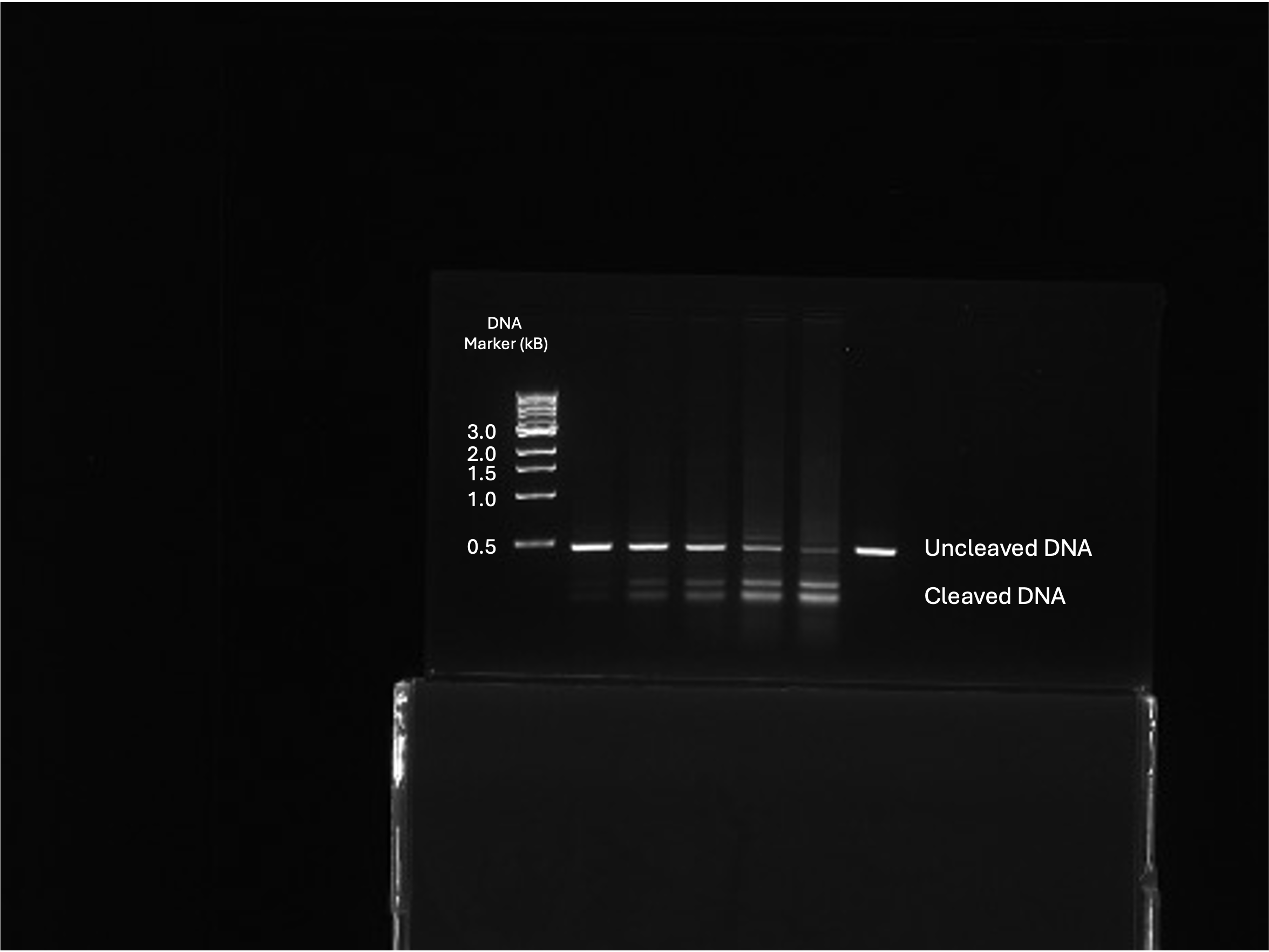

Supplement: Figure 7—figure supplement 1—source data 2. [file elife-99275-fig7-figsupp1-data2.zip › Figure 7 - figure supplement 1 - source data 2/R332A GeoCas9/R332A GeoCas9 at 37 ┬░C.png]

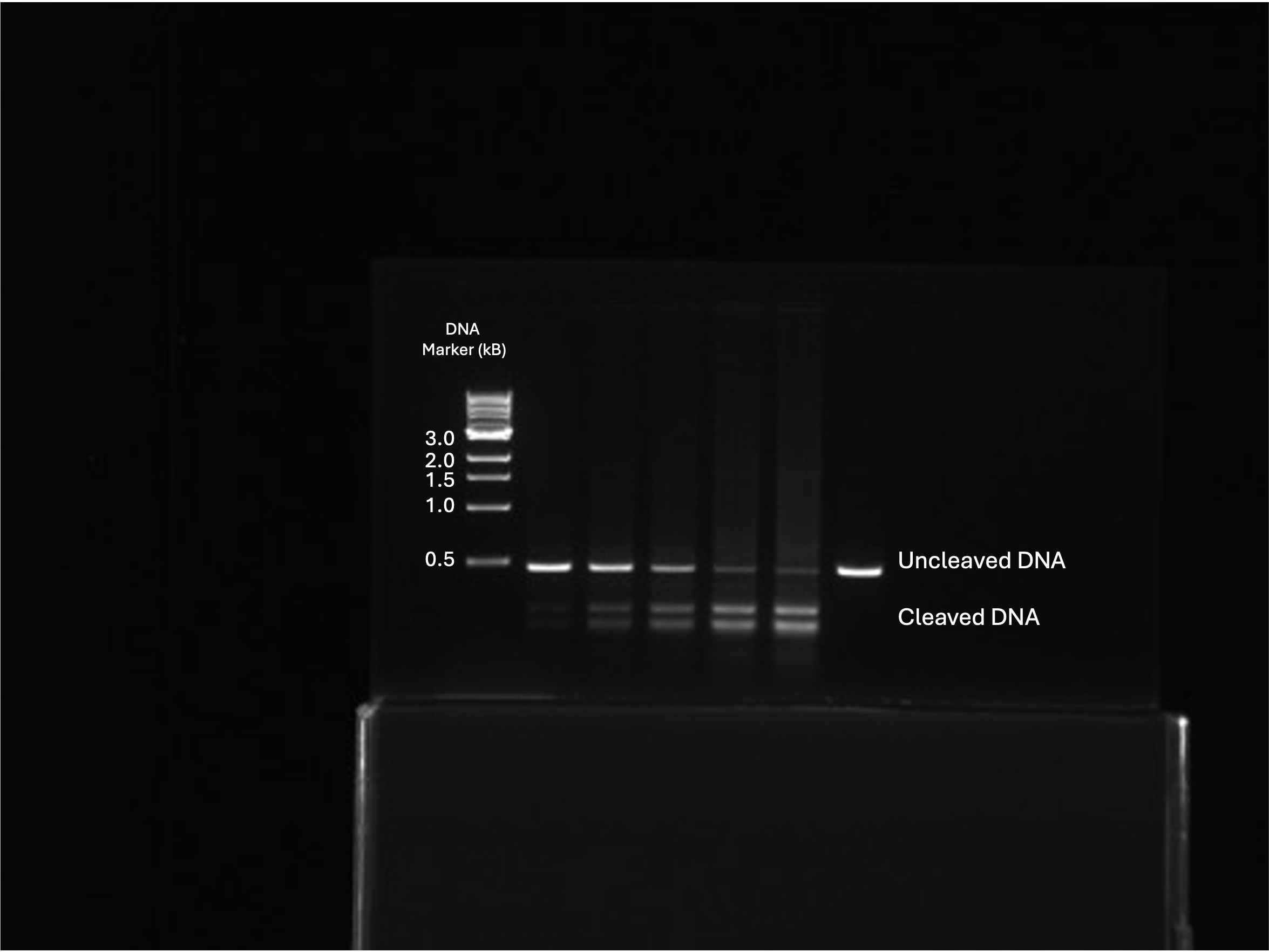

Supplement: Figure 7—figure supplement 1—source data 2. [file elife-99275-fig7-figsupp1-data2.zip › Figure 7 - figure supplement 1 - source data 2/R332A GeoCas9/R332A GeoCas9 at 60 ┬░C.png]

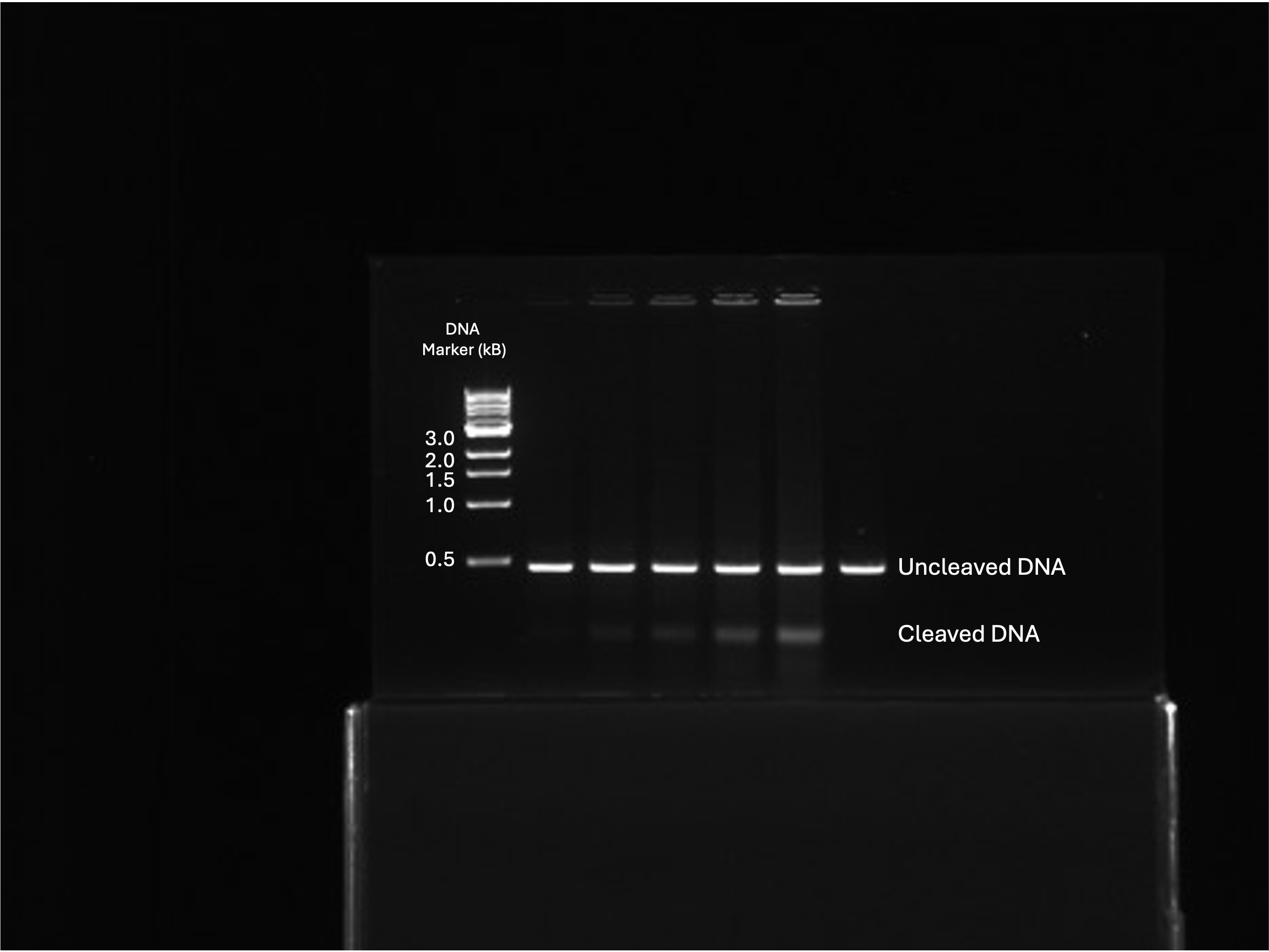

Supplement: Figure 7—figure supplement 1—source data 2. [file elife-99275-fig7-figsupp1-data2.zip › Figure 7 - figure supplement 1 - source data 2/R332A GeoCas9/R332A GeoCas9 at 75 ┬░C.png]

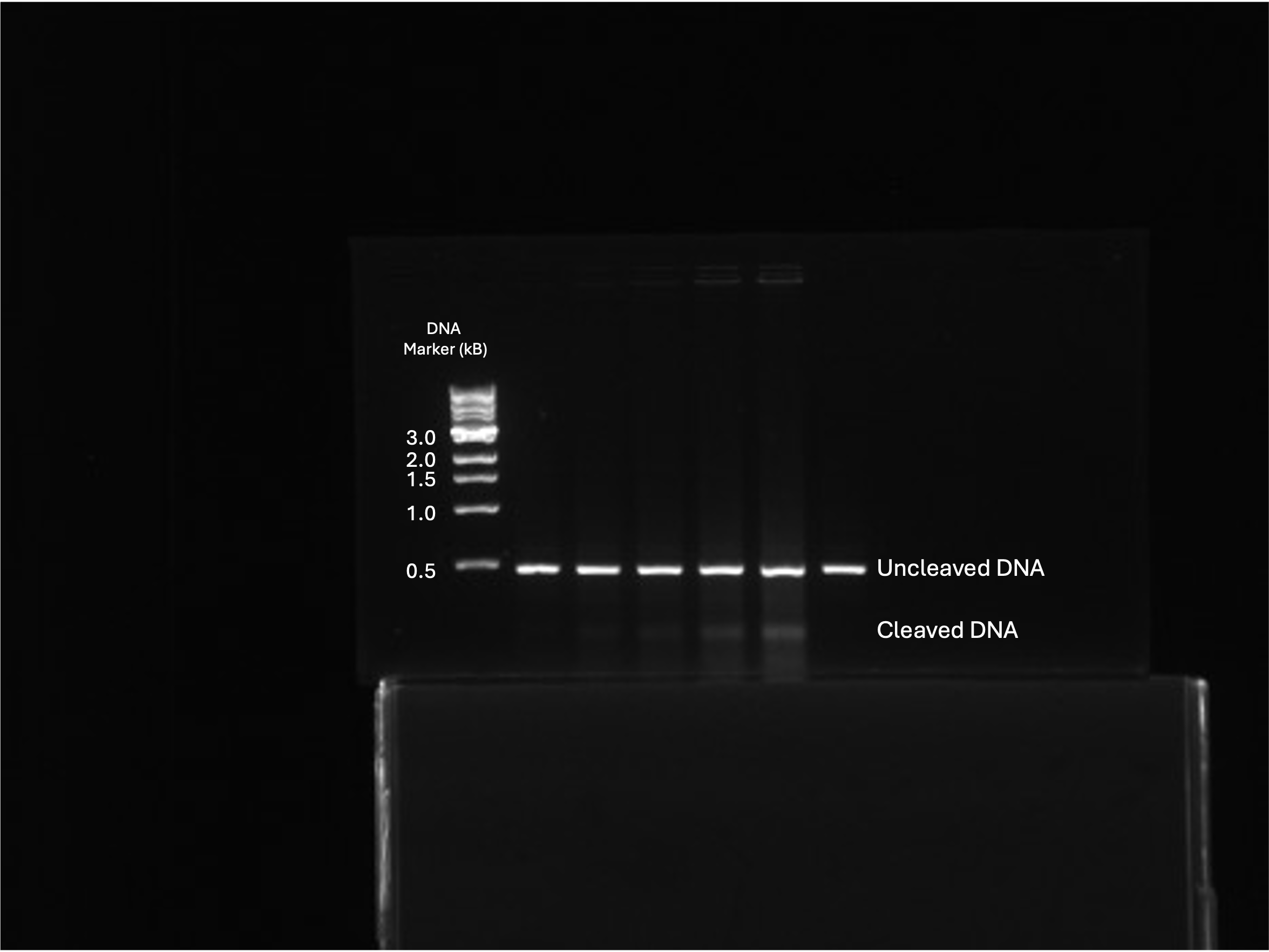

Supplement: Figure 7—figure supplement 1—source data 2. [file elife-99275-fig7-figsupp1-data2.zip › Figure 7 - figure supplement 1 - source data 2/K267E GeoCas9/K267E GeoCas9 at 85 ┬░C.png]

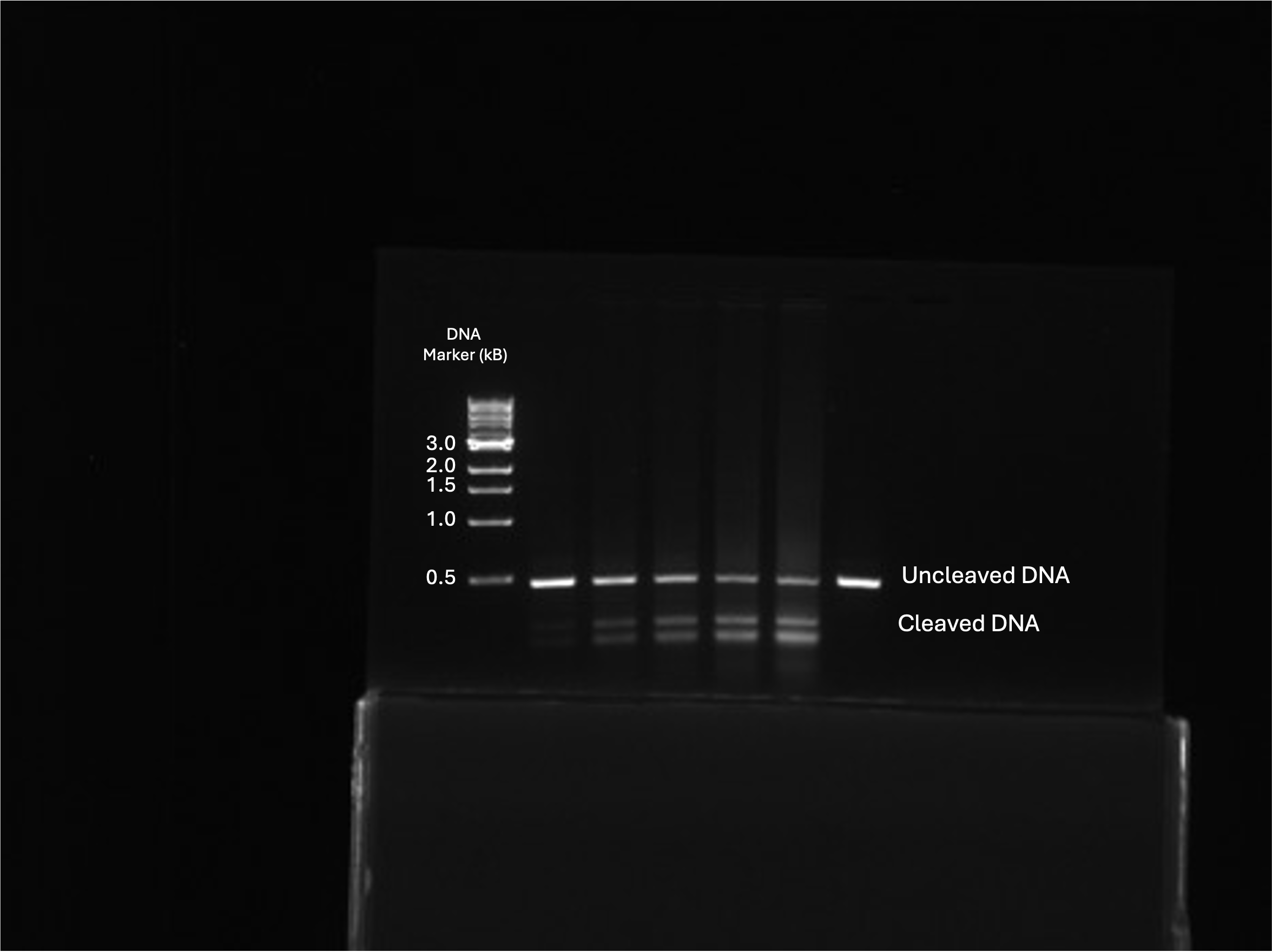

Supplement: Figure 7—figure supplement 1—source data 2. [file elife-99275-fig7-figsupp1-data2.zip › Figure 7 - figure supplement 1 - source data 2/K267E GeoCas9/K267E GeoCas9 at 60 ┬░C.png]

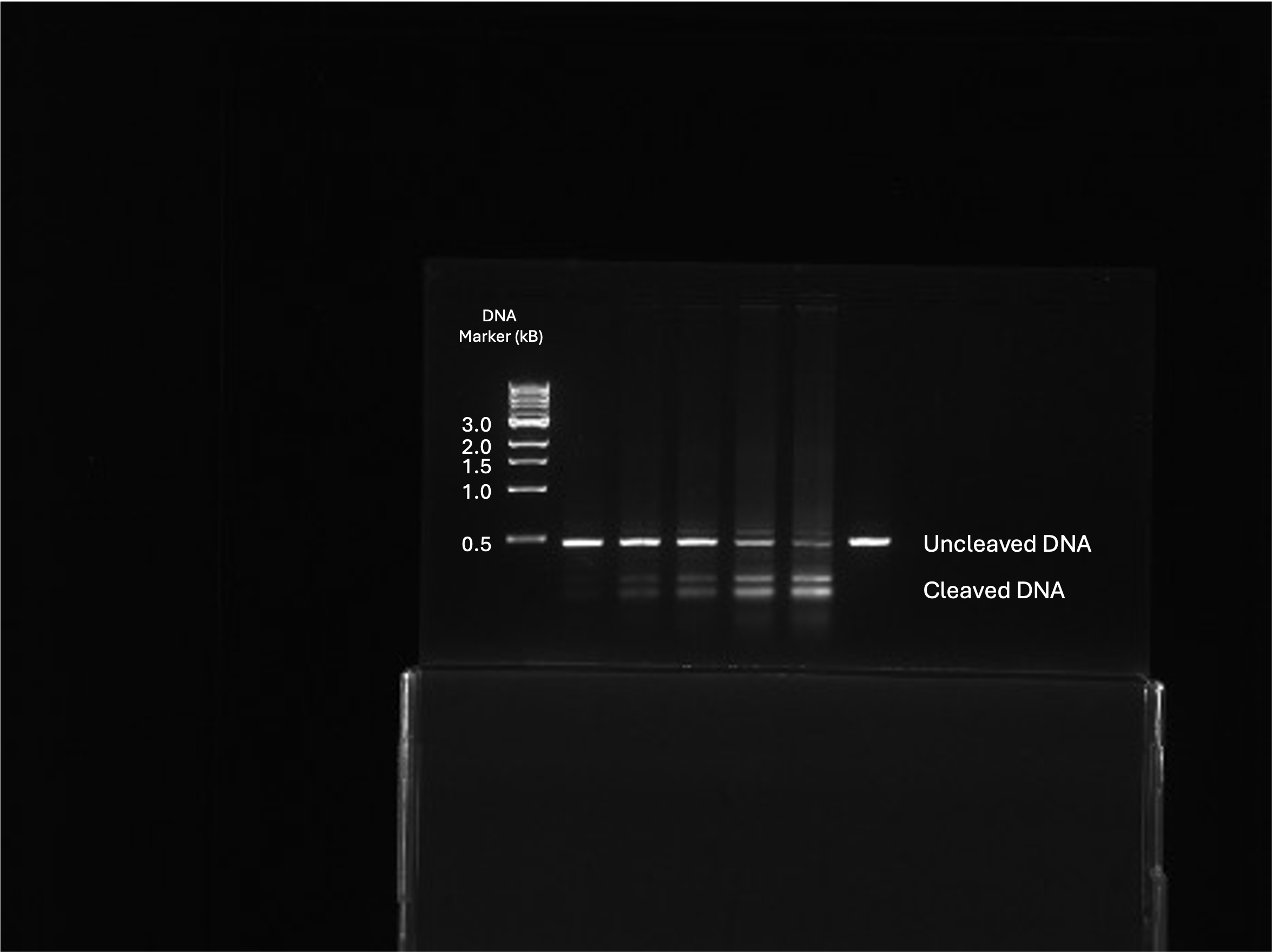

Supplement: Figure 7—figure supplement 1—source data 2. [file elife-99275-fig7-figsupp1-data2.zip › Figure 7 - figure supplement 1 - source data 2/K267E GeoCas9/K267E GeoCas9 at 37 ┬░C.png]

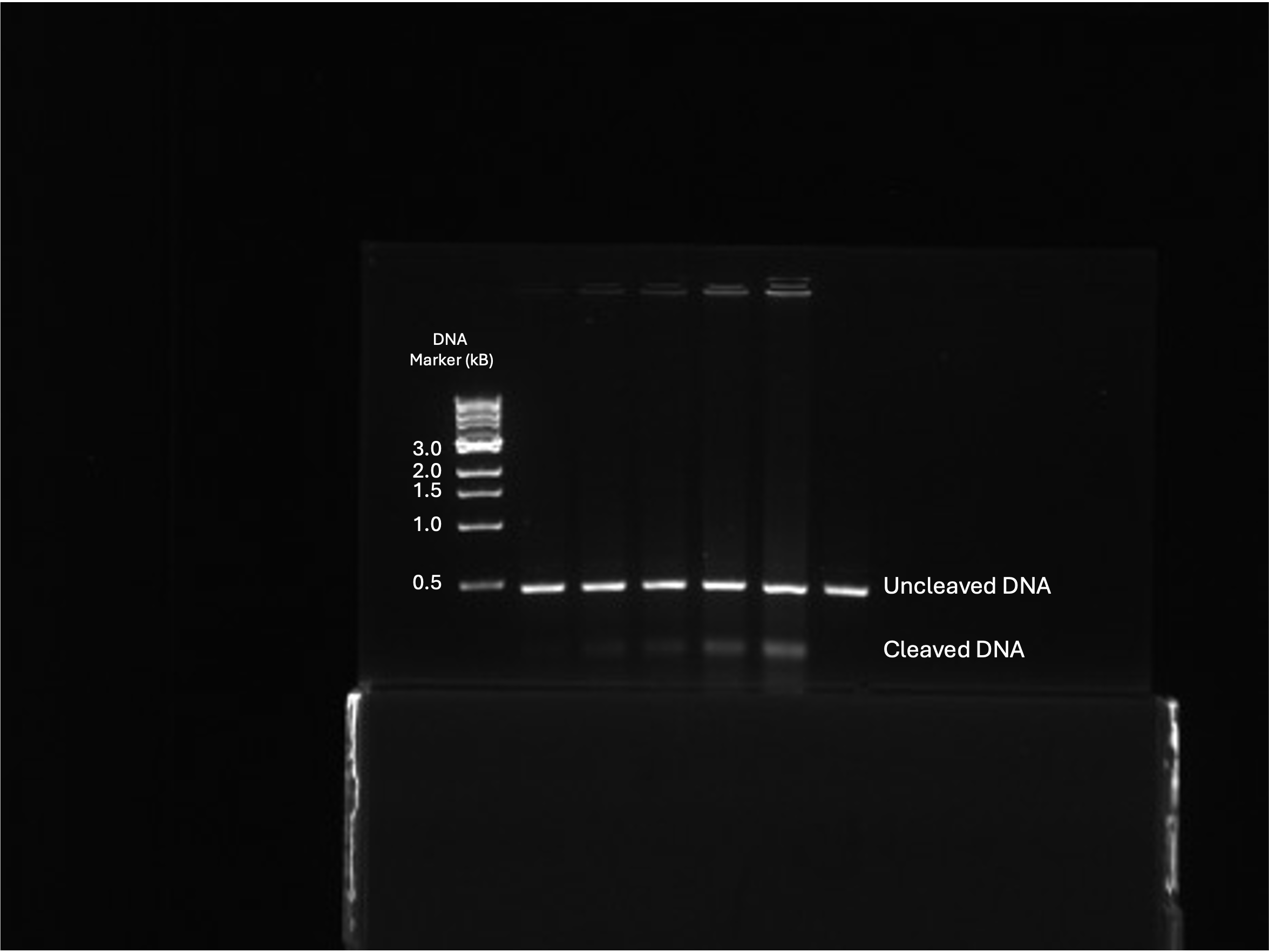

Supplement: Figure 7—figure supplement 1—source data 2. [file elife-99275-fig7-figsupp1-data2.zip › Figure 7 - figure supplement 1 - source data 2/K267E GeoCas9/K267E GeoCas9 at 75 ┬░C.png]
