# Supplementary material for: Structural and dynamic impacts of single-atom disruptions to guide RNA interactions within the recognition lobe of Geobacillus stearothermophilus Cas9
Source: eLife. 2025 May 19;13:RP99275. doi: 10.7554/eLife.99275 (PMC12088677; doi:10.7554/eLife.99275)
Supplement: Supplementary file 1. — A very small number of other resonances displaying curved CPMG profiles could not be globally fit and were excluded from this list. In all samples (i.e. WT, K267E, and R332A), the global fit was found to be the best statistical model. [file elife-99275-supp1.docx]

| Wild-type | K267E | | R332A |
| --- | --- | --- | --- |
| k_ex_ = 147 ± 41 s^-1^ | k_ex_ = 376 ± 89 s^-1^ | | k_ex_ = 142 ± 28 s^-1^ |
| **F256** | **A249** | **V349** | **Q257** |
| **N266** | **I265** | **L359** | **G275** |
| **K267** | **L268** | **D362** | **A276** |
| **L270** | **L270** | **F363** | **R277** |
| **G275** | **I271** | **D376** | **L286** |
| **A276** | **S272** | **E388** | **T312** |
| **I297** | **G275** | **N399** | **Y319** |
| **D311** | **D281** | **N404** | **K326** |
| **Y313** | **L286** | **L406** | **N330** |
| **K315** | **K296** | **N412** | **F333** |
| **Y319** | **D310** | **L413** | **K344** |
| **K354** | **Y313** | **E434** | **V349** |
| **S356** | **Y319** | **V438** | **L359** |
| **Y387** | **E329** | **R445** | **D376** |
| **E409** | **N330** |  | **M394** |
| **L413** | **F333** |  | **D403** |
| **I429** | **E335** |  | **N412** |
|  | **R343** |  | **L413** |

**Supplementary File 1.** Residues fit to a global *k*_ex_ in ^1^H-^15^N CPMG relaxation dispersion analysis of WT, K267E, and R332A *Geo*Rec2. A very small number of other resonances displaying curved CPMG profiles could not be globally fit and were excluded from this list. In all samples (*i.e.* WT, K267E, and R332A), the global fit was found to be the best statistical model.

.
