# Supplementary material for: Structural and dynamic impacts of single-atom disruptions to guide RNA interactions within the recognition lobe of Geobacillus stearothermophilus Cas9
Source: eLife. 2025 May 19;13:RP99275. doi: 10.7554/eLife.99275 (PMC12088677; doi:10.7554/eLife.99275)
Supplement: Supplementary file 2. — The 39-nucleotide sequence of RNA used in MST and NMR studies of isolated GeoRec is underlined. [file elife-99275-supp2.docx]

| Tnnt2 gRNA | UUGCACCUACCUUCUGGAUGUACGUCAUAGUUCCCCUGAGAAAUCAGGGUUACUAUGAUAAGGGCUUUCUGCCUAAGGCAGACUGACCCGCGGCGUUGGGGAUCGCCUGUCGCCCGCUUUUGGCGGGCAUUCCCCAUCCUU |
| --- | --- |
| 8UZA gRNA | CACUGCAUUCUAGUUGUGGUUGUCAUAGUUCCCCUGAGAAAUCAGGGUUACUAUGAUAAGGGCUUUCUGCCUAAGGCAGACUGACCCGCGGCGUUGGGGAUCGCCUGUCGCCCGCUUUUGGCGGGCAUUCCCCAUCCUU |

**Supplementary File 2.** Guide RNA sequences used in *Geo*Cas9 MST measurements. The 39-nucleotide sequence of RNA used in MST and NMR studies of isolated *Geo*Rec is underlined.
