# Supplementary material for: Structural and dynamic impacts of single-atom disruptions to guide RNA interactions within the recognition lobe of Geobacillus stearothermophilus Cas9
Source: eLife. 2025 May 19;13:RP99275. doi: 10.7554/eLife.99275 (PMC12088677; doi:10.7554/eLife.99275)
Supplement: Supplementary file 3. — The 23 base pair spacer sequence of gRNA is underlined. The spacer sequence within the DNA sequences is highlighted yellow, and the PAM are highlighted in blue. [file elife-99275-supp3.docx]

| sgRNA | UUGCACCUACCUUCUGGAUGUACGUCAUAGUUCCCCUGAGAAAUCAGGGUUACUAUGAUAAGGGCUUUCUGCCUAAGGCAGACUGACCCGCGGCGUUGGGGAUCGCCUGUCGCCCGCUUUUGGCGGGCAUUCCCCAUCCUU |
| --- | --- |
| On-target DNA | CAAAGAGCTCCTCGTCCAGTGGGAAGAGAGCTGATCTCATTTGTAAGGAATACCCTCTTCATCCCCCACCCTTGCCATTGATCTATTCATTCCATCTCCATGACAACAGGAAGAGAGGGCCCGGCGTGAGGAGGAGGAGAACAGGAGGAAGGCTGAGGATGAGGCCCGGAAGAAGAAGGCTCTGTCCAACATGATGCACTTTGGAGGGTACATCCAGAAGGTAGGTGCAAAGCAGCATCGGGCACCAGGACACCCCAGTGTATCCTCAAGGCCGCCTTTGCTTGGATCCATGAAGAAATTCCCAACTGCTGGTGGCTGAAGTCTAAGGTCTGCTCATGTCTAGCCCCTGAGCTGTCTATCAGCCTGACCATGGTTCAGTAGGAGGGCTCTGCTGTGTGTGACAGTTAGAACACTAATATGTCTCCAAATTCTGGCTCCCCAAAGGGACAACTGGGAGAATCTTGGGTCCTGGAGTCCAT |
| Off-target DNA  PAM proximal mismatch (5-6 bp AT 🡪 CA) | CAAAGAGCTCCTCGTCCAGTGGGAAGAGAGCTGATCTCATTTGTAAGGAATACCCTCTTCATCCCCCACCCTTGCCATTGATCTATTCATTCCATCTCCATGACAACAGGAAGAGAGGGCCCGGCGTGAGGAGGAGGAGAACAGGAGGAAGGCTGAGGATGAGGCCCGGAAGAAGAAGGCTCTGTCCAACATGATGCACTTTGGAGGGTACcaCCAGAAGGTAGGTGCAAAGCAGCATCGGGCACCAGGACACCCCAGTGTATCCTCAAGGCCGCCTTTGCTTGGATCCATGAAGAAATTCCCAACTGCTGGTGGCTGAAGTCTAAGGTCTGCTCATGTCTAGCCCCTGAGCTGTCTATCAGCCTGACCATGGTTCAGTAGGAGGGCTCTGCTGTGTGTGACAGTTAGAACACTAATATGTCTCCAAATTCTGGCTCCCCAAAGGGACAACTGGGAGAATCTTGGGTCCTGGAGTCCAT |
| Off-target DNA  PAM distal mismatch (19-20 bp TG 🡪 CA) | CAAAGAGCTCCTCGTCCAGTGGGAAGAGAGCTGATCTCATTTGTAAGGAATACCCTCTTCATCCCCCACCCTTGCCATTGATCTATTCATTCCATCTCCATGACAACAGGAAGAGAGGGCCCGGCGTGAGGAGGAGGAGAACAGGAGGAAGGCTGAGGATGAGGCCCGGAAGAAGAAGGCTCTGTCCAACATGATGCACTTTGGAGGGTACATCCAGAAGGTAGGcaCAAAGCAGCATCGGGCACCAGGACACCCCAGTGTATCCTCAAGGCCGCCTTTGCTTGGATCCATGAAGAAATTCCCAACTGCTGGTGGCTGAAGTCTAAGGTCTGCTCATGTCTAGCCCCTGAGCTGTCTATCAGCCTGACCATGGTTCAGTAGGAGGGCTCTGCTGTGTGTGACAGTTAGAACACTAATATGTCTCCAAATTCTGGCTCCCCAAAGGGACAACTGGGAGAATCTTGGGTCCTGGAGTCCAT |

**Supplementary File 3.** Nucleic acid sequences used in the *Geo*Cas9 *in vitro* off-target assay. The 23 base pair spacer sequence of gRNA is underlined. The spacer sequence within the DNA sequences is highlighted yellow, and the PAM are highlighted in blue.
