# Supplementary material for: Structural and dynamic impacts of single-atom disruptions to guide RNA interactions within the recognition lobe of Geobacillus stearothermophilus Cas9
Source: eLife. 2025 May 19;13:RP99275. doi: 10.7554/eLife.99275 (PMC12088677; doi:10.7554/eLife.99275)
Supplement: Supplementary file 4. — Sites of mismatched DNA are highlighted in red. [file elife-99275-supp4.docx]

| On-target DNA | CAAAGAGCTCCTCGTCCAGTGGGAAGAGAGCTGATCTCATTTGTAAGGAATACCCTCTTCATCCCCCACCCTTGCCATTGATCTATTCATTCCATCTCCATGACAACAGGAAGAGAGGGCCCGGCGTGAGGAGGAGGAGAACAGGAGGAAGGCTGAGGATGAGGCCCGGAAGctGAAGGCTCTGTCCAACATGATGCACTTTGGAGGGTACATCCAGAAGGTAGGTGCAAAGCAGCATCGGGCACCAGGACACCCCAGTGTATCCTCAAGGCCGCCTTTGCTTGGATCCATGAAGAAATTCCCAACTGCTGGTGGCTGAAGTCTAAGGTCTGCTCATGTCTAGCCCCTGAGCTGTCTATCAGCCTGACCATGGTTCAGTAGGAGGGCTCTGCTGTGTGTGACAGTTAGAACACTAATATGTCTCCAAATTCTGGCTCCCCAAAGGGACAACTGGGAGAATCTTGGGTCCTGGAGTCCAT |
| --- | --- |
| Off-target DNA  PAM proximal mismatch (5-6 bp AA 🡪 CT) | CAAAGAGCTCCTCGTCCAGTGGGAAGAGAGCTGATCTCATTTGTAAGGAATACCCTCTTCATCCCCCACCCTTGCCATTGATCTATTCATTCCATCTCCATGACAACAGGAAGAGAGGGCCCGGCGTGAGGAGGAGGAGAACAGGAGGAAGGCTGAGGATGAGGCCCGGAAGAAGAAGGCTCTGTCagACATGATGCACTTTGGAGGGTACATCCAGAAGGTAGGTGCAAAGCAGCATCGGGCACCAGGACACCCCAGTGTATCCTCAAGGCCGCCTTTGCTTGGATCCATGAAGAAATTCCCAACTGCTGGTGGCTGAAGTCTAAGGTCTGCTCATGTCTAGCCCCTGAGCTGTCTATCAGCCTGACCATGGTTCAGTAGGAGGGCTCTGCTGTGTGTGACAGTTAGAACACTAATATGTCTCCAAATTCTGGCTCCCCAAAGGGACAACTGGGAGAATCTTGGGTCCTGGAGTCCAT |
| Off-target DNA  PAM distal mismatch (19-20 bp CA 🡪 AG) | CAAAGAGCTCCTCGTCCAGTGGGAAGAGAGCTGATCTCATTTGTAAGGAATACCCTCTTCATCCCCCACCCTTGCCATTGATCTATTCATTCCATCTCCATGACAACAGGAAGAGAGGGCCCGGCGTGAGGAGGAGGAGAACAGGAGGAAGGCTGAGGATGAGGCCCGGAAGAAGAAGGCTCTGTCCAACATGATGCACTTTGGAGGGTACATCCAGAAGGTAGGTGCAAAGCAGCATCGGGCACCAGGACACCCCAGTGTATCCTCAAGGCCGCCTTTGCTTGGATCCATGAAGAAATTCCCAACTGCTGGTGGCTGAAGTCTAAGGTCTGCTCATGTCTAGCCCCTGAGCTGTCTATCAGCCTGACCATGGTTCAGTAGGAGGGCTCTGCTGTGTGTGACAGTTAGAACACTAATATGTCTCCAAATTCTGGCTCCCCAAAGGGACAACTGGGAGAATCTTGGGTCCTGGAGTCCAT |

**Supplementary File 4.** DNA sequences used in the *Sp*Cas9 *in vitro* off-target assay. Sites of mismatched DNA are highlighted in red.
